# Apolipoprotein E levels in the amygdala and prefrontal cortex predict relative regional brain volumes in irradiated Rhesus macaques

**DOI:** 10.1038/s41598-021-01480-3

**Published:** 2021-11-11

**Authors:** Payel Kundu, Benjamin Zimmerman, Ruby Perez, Christopher T. Whitlow, J. Mark Cline, John D. Olson, Rachel N. Andrews, Jacob Raber

**Affiliations:** 1 Department of Behavioral Neuroscience, Oregon Health and Science University, Portland, OR USA; 2 Advanced Imaging Research Center, Oregon Health and Science University, Portland, OR USA; 3 Beckman Institute for Advanced Science and Technology, University of Illinois at Urbana-Champaign, Urbana, IL USA; 4 Department of Radiology, Radiology Informatics & Image Processing Laboratory (RIIPL), Wake Forest University, Wake Forest University School of Medicine, Winston-Salem, NC USA; 5 Department of Pathology, Section on Comparative Medicine, Wake Forest University School of Medicine, Winston-Salem, NC USA; 6 Division of Neuroscience, Departments of Neurology and Radiation Medicine, ONPRC, Oregon Health and Science University, Portland, OR USA

**Keywords:** Neuroscience, Biomarkers

## Abstract

In the brain, apolipoprotein E (apoE) plays an important role in lipid transport and response to environmental and age-related challenges, including neuronal repair following injury. While much has been learned from radiation studies in rodents, a gap in our knowledge is how radiation might affect the brain in primates. This is important for assessing risk to the brain following radiotherapy as part of cancer treatment or environmental radiation exposure as part of a nuclear accident, bioterrorism, or a nuclear attack. In this study, we investigated the effects of ionizing radiation on brain volumes and apoE levels in the prefrontal cortex, amygdala, and hippocampus of Rhesus macaques that were part of the Nonhuman Primate Radiation Survivor Cohort at the Wake Forest University. This unique cohort is composed of Rhesus macaques that had previously received single total body doses of 6.5–8.05 Gy of ionizing radiation. Regional apoE levels predicted regional volume in the amygdala and the prefrontal cortex. In addition, apoE levels in the amygdala, but not the hippocampus, strongly predicted relative hippocampal volume. Finally, radiation dose negatively affected relative hippocampal volume when apoE levels in the amygdala were controlled for, suggesting a protective compensatory role of regional apoE levels following radiation exposure. In a supplementary analysis, there also was a robust positive relationship between the neuroprotective protein α-klotho and apoE levels in the amygdala, further supporting the potentially protective role of apoE. Increased understanding of the effects of IR in the primate brain and the role of apoE in the irradiated brain could inform future therapies to mitigate the adverse effects of IR on the CNS.

## Introduction

Exposure to ionizing radiation (IR) can cause DNA damage, potentially resulting in single- and double-strand breaks, base and nucleotide damages, and DNA and protein crosslinks^1^. In addition, radiation can lead to changes in gene expression, regulation of the cell cycle, and global genome stability^2^. At doses lower than those needed to induce acute radiation syndrome, multiple studies have indicated that there is a risk of developing adverse delayed effects involving inflammatory and degenerative conditions in multiple organ systems^3^. This risk is particularly relevant to the treatment of cancer, since over half of cancer patients undergo radiation therapy in some form^4^. High-dose cranial radiation therapy is associated with numerous adverse central nervous system (CNS) effects, including declines in cognitive function^4^. Lower radiation doses can also cause detectable impairments in cognitive function in the absence of apparent morphological changes^5^. The pathogenesis of radiation-induced cognitive injury, especially at low doses, is not fully known. In rodents, ionizing radiation inhibits adult neurogenesis by killing neural precursor cells in the subgranular zone of the hippocampus in a dose-dependent fashion, and is also associated with a chronic inflammatory response, as measured by an increase in activated microglia^5,6^. Neuronal precursor cells are extremely sensitive to radiation and undergo apoptosis even at clinically relevant doses (0.5–2.0 Gy) that do not cause evident tissue injury^7,8^. These effects are long lasting, resulting in reduced neurogenesis as well as impaired spatial memory 3 months after radiation exposure^6^.

Radiation may also accelerate pathological as well as age-related cognitive decline^9^. IR increases oxidative stress and neuroinflammation through disruption of mitochondria, this pathology overlaps with many neurodegenerative diseases such as Alzheimer’s disease (AD) and Parkinson’s disease (PD)^9^. In mouse models of AD (APP/PS1) receiving exposure to IR at a similar cumulative dose as what astronauts are exposed to in deep space, irradiated mice developed cognitive impairment and increased amyloid-β (Aβ) plaque formation 6 months after exposure compared to sham-irradiated mice^10,11^. Increased Aβ plaque formation might involve altered Aβ trafficking through the blood–brain-barrier and altered clearance^11^. This is supported by the finding that IR can increase the permeability of the BBB^12^. There is scant research on the possibility that IR exacerbates pathology in neurodegenerative diseases, and what research does exist has been conducted in rodent models.

Nonhuman primates (NHPs) are considered the gold standard of animal models due to greater homology with humans in organ structure, genetics, and lifespan^13^*.* NHPs exhibit naturally occurring age-related Aβ depositions in brain parenchyma as well as vasculature, making them excellent models for studying human neurodegenerative disease^14,15^. Aging rhesus macaques exhibit age-related cognitive deficits, alterations in circadian activity that are associated with cognitive impairments, immune-related measures, amyloid plaque pathology, as well as tau pathology similar in qualitative pattern and temporal sequence as humans^16–21^.

Apolipoprotein E (apoE) plays an important role in lipid transport as well as neuronal repair following injury^16^. Humans carry three alleles for apoE. Compared to apoE3, apoE4 increases the risk of developing Alzheimer disease and cognitive impairments following various environmental challenges such as high fat diet^17,18^. Homozygous apoE4 carriers have lower brain levels of apoE in the hippocampus, frontal cortex and cerebrospinal fluid (CSF) compared to homozygous apoE3 or apoE2 levels^19,22,23^. Thus homozygous E2 carriers have the highest apoE levels in the CNS, while homozygous E4 carriers have the lowest. ApoE isoform also modulates radiation-induced cognitive impairment; mice lacking apoE are more susceptible than wild-type mice to the effects of ^56^Fe ion irradiation on motor coordination, exploratory activity, and spatial working memory^16^. Thus, apoE might play a protective role against radiation-induced cognitive injury. Additionally, apoE isoform modulates the direction of ^56^Fe irradiation-induced spatial memory deficits in mice in a sex-dependent manner^24^. Levels of apoE are increased in the aged nonhuman primate brain and in the mouse brain response to environmental challenges such as irradiation^25–27^*.* An unanswered question is whether apoE regulation plays an adaptive role in protecting the brain from environmental challenges such as IR. While low apoE levels in the brain are associated with poorer cognitive outcomes, it is not clear if this is secondary to more apoE bound in plaques in those individuals, or if low apoE levels are actually contributing to the cognitive decline because of the potentially protective role of apoE-mediated synaptic repair and Aβ clearance in the brain^22,28^. Outcomes may also depend on the cellular origin of the apoE in brain.

The Klotho gene encodes a single-pass transmembrane protein and contains three subfamilies: α-klotho, β-klotho, and γ-klotho^29,30^. Klotho, specifically α-klotho, may play a role in neurodegenerative diseases and cognitive aging. Transgenic mice with systemic overexpression of human α-klotho perform better than control mice on a variety of cognitive measures, including spatial and working memory as well as fear memory^31^. In a meta review of collectively 718 human individuals, heterozygote carriers of the KL-VS klotho variant scored higher on a composite test of global cognition than non-carriers^31^. Heterozygote carriers of the KL-VS klotho variant have two- to three-fold higher serum levels of α-klotho proteins than non-carriers^31,32^. KL-VS heterozygosity was also associated with larger right dorsolateral prefrontal cortex volume and enhanced executive function compared to non KL-VS carriers in a sample of cognitively normal older adults from two independent cohorts^33^. α-klotho protein levels in the CSF are lower in older adults with Alzheimer’s disease (AD) compared to age matched controls, and decline with age^34^. Thus, higher levels of α-klotho are associated with neuroprotection against cognitive and volumetric loss.

Whole brain volume, both white and gray matter, decline linearly over the lifespan in nondemented individuals. With Alzheimer’s-like dementia, that rate is markedly accelerated^35^. It is known that apoE genotype has strong effects on processes in normal brains, as well as the propensity to develop AD^23^. However, it is unclear how apoE levels affect regional brain volumes. The literature on the effects of apoE genotype on regional brain volume in young adults is mixed, with some literature indicating limited effects in the entorhinal cortex, and others indicating no relationship^22,23,36^. However, in healthy adults over 60 years of age, apoE4 carriers had significantly smaller hippocampal and amygdala volumes than non-E4 carriers^37^. Whether this is mediated by brain levels of apoE is unknown and harder to separate out in humans with both levels and apoE isoforms being different in individuals.

The Radiation Survivor cohort at Wake Forest is composed of Rhesus macaques previously exposed to single total body doses of 3.5–8.4 Gy of ionizing radiation at the following institutions: the Armed Forces Radiobiology Research Institute (AFRRI), the University of Maryland (UMD), the University of Illinois at Chicago (UIC), Harvard, and the Wake Forest School of Medicine. In contrast to the human scenario, in this unique resource, the apoE isoform is the same across all individuals^38^. In the present study, we investigated whether brain apoE change in response to IR in Rhesus macaques, as well as whether there is a relationship between regional apoE levels and brain volume in the hippocampus, amygdala, and prefrontal cortex (PFC). We selected these brain regions as they are relevant to cognition and anxiety. We hypothesized, based on our previous mouse data, that irradiation may cause premature aging, as assessed by regional brain volumes, and relate to the variance in regional apoE levels in the brain^25,26^. As a supplementary analysis, we investigated whether levels of α-klotho in the amygdala or PFC were associated with any of our outcome measures.

## Methods

### Subjects

Animals were selected from the Wake Forest Non-Human Primate Radiation Survivor Cohort. The cohort is comprised of Rhesus macaques who survived ionizing radiation studies performed at other institutions. To be selected for this study animals had to be deceased and have had brain tissue harvested at necropsy and had to have had an MRI scan prior to death. At the time of animal selection, the cohort was 75% male, so the animals from the cohort who met selection criteria for inclusion in this study happened to be all male. The age of death of the animals varied between 8 and 19 years (*M* = 12.74, *SD* = 3.06).

All in vivo blood collections and other post-irradiation procedures were conducted at the Wake Forest University School of Medicine with approval by the Institutional Animal Care and Use Committee of Wake Forest University. Wake Forest University is committed to providing a high-quality program of animal care in compliance with state and federal Animal Welfare Acts and the standards and policies of the US Department of Health and Human Services. Wake Forest University has an Assurance on file in the Office for Protection from Research Risks, Office of the Director, National Institutes of Health, that accepts responsibility for the humane care and use of animals (OPRR #A-3391-01). The Laboratory Animal Care Program of the Wake Forest University School of Medicine complies with the "Principles for Use of Animals", the "Guide for the Care and Use of Laboratory Animals^39^", all provisions of the Animal Welfare Act, and has been accredited by the Association for Assessment and Accreditation of Laboratory Animal Care, International (AAALAC) since April 8, 1966 (AAALAC File #8).

**Figure 1:**
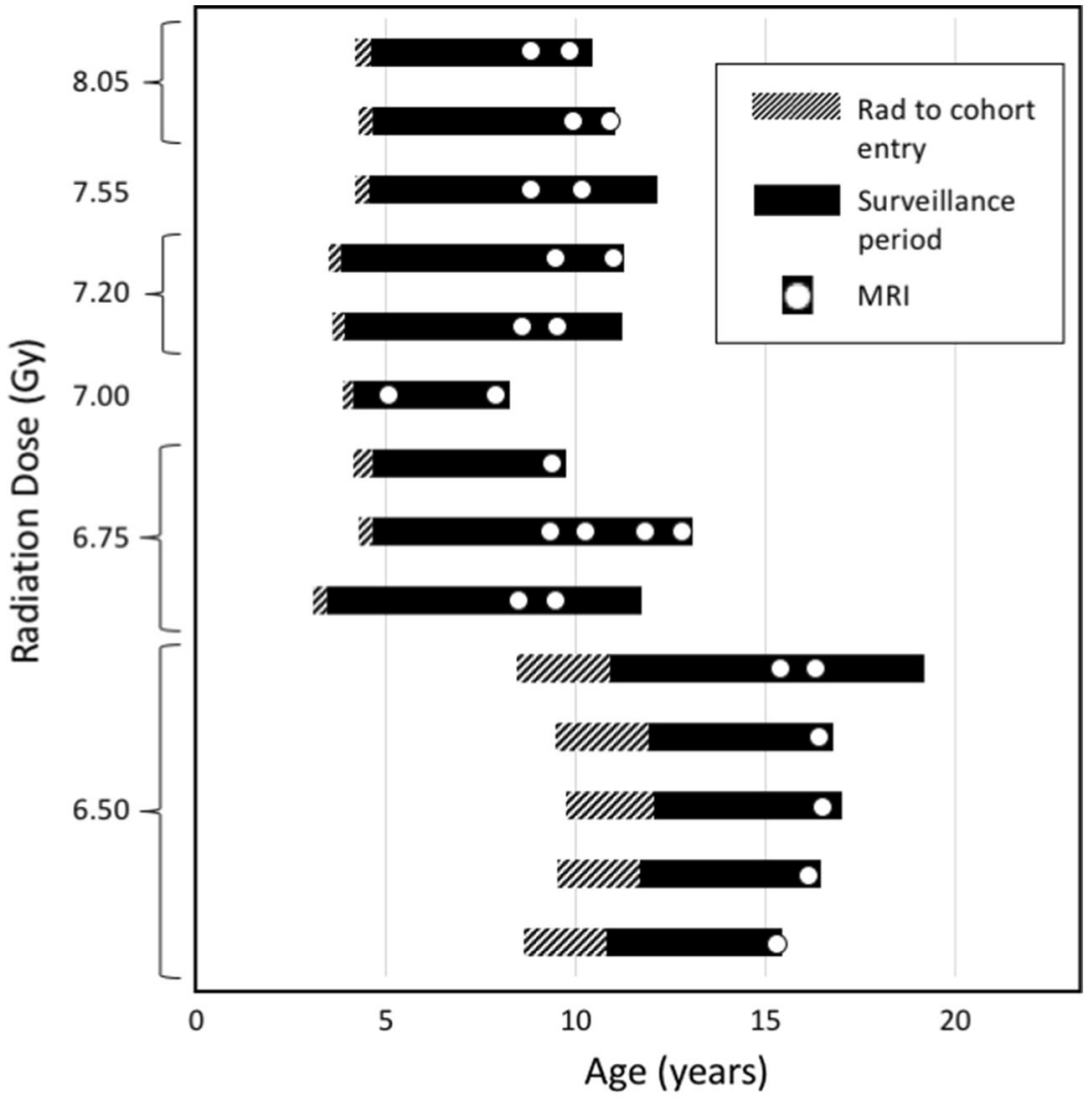
Timeline illustrating the time between radiation exposure and entry into the study, as well as the timing and number of MRI scans for each monkey.

The irradiated animals used for this study were obtained from University of Maryland, University of Illinois, and Armed Forces Radiobiological Research Institute. Irradiated animals received 6.5 to 8.05 Gy, median dose of 6.75 Gy, total body radiation under IACUC oversight at their prior institution using one of two strategies: (1) linear accelerator-derived photons at a nominal mean energy of 2 MeV, delivered at 80 cGy/minute as a split dose given half anterior–posterior and half posterior-anterior; or (2) Cobalt 60-derived gamma irradiation delivered simultaneously, bilaterally at 60 cGy/min. Note that these are potentially lethal doses: the LD 10/30 for rhesus macaques is ~ 5.5 Gy, the LD 50/30 is ~ 6.7 Gy, and the LD 90/30 is 8.0 Gy^40^. The animals were irradiated at ages varying between 36 and 117 months old (*M* = 62.14 months, *SD* = 27.76 months). Surviving animals were subsequently transferred to Wake Forest School of Medicine Center for Comparative Medicine Research for long-term monitoring post-radiation. Irradiation methods, supportive care strategies, and acute effects for many animals donated to this cohort have been recently reported^40–42^. The timing between radiation exposure and entry into the study, as well as the timeline of MRI scans is shown in Fig. 1.

After arrival at Wake Forest, all animals were monitored twice daily by trained veterinary technical laboratory staff to assure animal well-being and social stability. Any evidence of illness was classified using a nonhuman primate-specific modification of the Children’s Clinical Oncology Group toxicity criteria^43^. Sick animals were promptly evaluated by one of seven institutional veterinarians independent of the research team, four of whom are board-certified by the American College of Laboratory Animal Medicine.

Animals were either fed commercially available monkey chow (Purina®, St. Louis, MO) or Typical American Primate diet (LabDiet 5L0P; Land O'Lakes Inc., St. Louis, MO] designed to approximate the macronutrients of a Western dietary profile. Their diet was supplemented with fresh fruits and vegetables and with water ad libitum. They were housed socially in indoor-outdoor pens whenever possible, or in group cages if necessary for safe handling or medical care. Care was taken to ensure the animals in the groups were compatible. Environmental enrichment, including fruits/vegetables, toys, puzzles, climbing and hiding environments, was provided continuously on a rotating basis. Behavioral well-being was additionally monitored and recommendations made as needed by an independent behavioral management team. All animals were trained to cooperate in handling procedures, to minimize stress. Sampling was scheduled so that the animals were sedated the minimum number of times required for data collection. All methods are reported in accordance with ARRIVE guidelines^44^.

At the time of necropsy, the animals were humanely euthanized in accordance with the American Veterinary Medical Association’s Guidelines on Euthanasia by deep anesthesia with pentobarbital, followed by exsanguination and perfusion of the vascular system with 2 L of cold normal saline. The brain was removed intact and sectioned coronally in 4-mm intervals using a stainless-steel brain matrix with cutting guides. Once removed from the matrix, all slices were photographed. Alternating sections were either immediately frozen on dry ice or immersed in 4% cold paraformaldehyde for 24 h. At the time of subdissection, the corresponding frozen coronal slabs were minimally thawed on dry ice, and the sub regions dissected by a board-certified veterinary pathologist with expertise in non-human primate neuroanatomy (RNA). The samples were then immediately re-frozen on dry ice and stored at − 80C until shipping.

### Quantification of ApoE levels

ApoE protein levels were quantified from lysates of available tissue from the hippocampus (13 animals), amygdala (12 animals) and PFC (14 animals)*.* The PFC samples were taken from Brodmann areas 9 and 46. The amount of tissue used from each region is as follows: hippocampus (0.04–0.09 g), amygdala (0.03–0.12 g), and PFC (0.05–0.13 g). These tissues are available as part of an archived tissue bank, with samples available on request. Limited sample was available for certain animals due to utilization in prior studies. Additionally, as alternating sections are fixed or frozen, due to minor inter-individual variation in anatomy, an ROI may not have been present within the frozen plane of section. Tissue was processed in ice cold buffer containing 1 M Tris–Cl, 6 M NaCl, 0.5 M EDTA, and Triton-X-100. Protein concentrations were determined using a BCA protein assay (Thermo Fisher, Waltham, MA). Levels of apoE were analyzed with an ELISA-based quantification assay (MyBioSource, San Diego, CA) according to the manufacturer’s instructions. Briefly, samples were diluted so that apoE protein levels fell within the range of the standard curve. Samples were then transferred to the 96-well ELISA plate. At the end of the assay, absorbances were read at 450 nm using an ID5 reader (Molecular Devices, San Jose, CA).

### Quantification of α-klotho levels

Levels of apoE increase with age, while levels of α-klotho decline^34,45^. To better understand the role of apoE, we included a supplementary analysis to investigate how α-klotho levels relate to levels of apoE. Protein levels were quantified from lysates of available tissue from the amygdala (12 animals) and the PFC (14 animals) using an ELISA-based quantification assay (IBL America, Minneapolis, MN). Briefly, test dilutions were conducted prior to full plate to ensure that α-klotho levels fell within the range of the standard curve. Samples were then transferred to the 96-well ELISA plate. At the end of the assay, absorbances were read at 450 nm using an ID5reader (Molecular Devices, San Jose, CA).

### Magnetic resonance imaging

#### MRI data acquisition

Animals were sedated with ketamine HCl (15 mg/kg body weight, IM) and maintained on inhaled isoflurane (3% induction, 1.5% maintenance) in 100% oxygen anesthesia for the duration of the ≤ 2 h long MRI procedure. One to four T1 weighted anatomical images were acquired annually for each macaque in the study. Because the selected animals entered the Delayed Effects of Radiation Cohort at different times, the imaging hardware, pulse sequences, and pulse sequence parameters were upgraded over the course of the study. Images were acquired on one of two 3 Tesla MRI scanners: a GE Signa Excite (GE Healthcare, Chicago, IL) or a Siemens Skyra (Seimens USA, Washington DC). Table 1 displays the scanners, coils, pulse sequences, and pulse sequence parameters used during the study. Table 2 displays the animal demographics and schedule of MRI scans.

**Table 1:** Imaging parameters for T1-anatomical images over different years.

Year	Scanner	Coil	Field strength	Pulse sequence	TR	TE	TI	Flip angle	Voxel size	FOV	Slice thickness	Matri × size
2012	GE Signa Excite	8 Ch Surface	3 T	3D SPGR	8.48 ms	3.59 ms	600 ms	15°	0.5 × 0.5mm^2^	128 × 128 mm^2^	0.5 mm	256 × 256
≥ 2013	Siemens Skyra	Body	3 T	MPRAGE	2700 ms	3.39 ms	880 ms	8°	0.5 × 0.5mm^2^	128 × 128 mm^2^	0.5 mm	256 × 256

**Table 2:** Animal demographics and MRI schedule.

ID	Origin^a^	Radiation dose (Gy)	Age at irradiation (years)	Age at necropsy (years)	MRI 1		MRI 2		MRI 3		MRI 4	
					Age at scan (years)	Post-rad interval (years)	Age at scan (years)	Post-rad interval (years)	Age at scan (years)	Post-rad interval (years)	Age at scan (years)	Post-rad interval (years)
NHP07	Chinese	6.5	8.6	15.4	15.3	6.7	NS	NS	NS	NS	NS	NS
NHP04	Chinese	6.5	9.5	16.4	16.2	6.7	NS	NS	NS	NS	NS	NS
NHP06	Indian	6.5	9.7	17.0	16.5	6.8	NS	NS	NS	NS	NS	NS
NHP03	Chinese	6.5	9.5	16.8	16.4	7.0	NS	NS	NS	NS	NS	NS
NHP05	Indian	6.5	8.4	19.2	15.4	7.0	16.3	7.9	NS	NS	NS	NS
NHP02	Chinese	6.75	3.1	11.7	8.3	5.3	9.3	6.2	NS	NS	NS	NS
NHP08	Chinese	6.75	4.3	13.1	9.2	4.9	10.1	5.8	11.7	7.4	12.7	8.4
NHP09	Chinese	6.75	4.1	9.7	9.2	5.0	NS	NS	NS	NS	NS	NS
NHP14	Chinese	7	3.8	8.3	4.8	0.9	7.7	3.8	NS	NS	NS	NS
NHP12	Chinese	7.2	3.6	11.2	8.4	4.8	9.3	5.8	NS	NS	NS	NS
NHP13	Chinese	7.2	3.5	11.3	9.3	5.8	10.8	7.4	NS	NS	NS	NS
NHP11	Chinese	7.55	4.2	12.1	8.6	4.4	10.0	5.9	NS	NS	NS	NS
NHP01	Chinese	8.05	4.3	11.0	9.7	5.5	10.8	6.5	NS	NS	NS	NS
NHP10	Chinese	8.05	4.2	10.4	8.6	4.4	9.7	5.5	NS	NS	NS	NS

^a^Origin refers to the genetic background of the animal.

*NS* not scanned.

#### MRI analysis

The analysis of the MR anatomical images involved a multi-step approach to ensure accurate non-linear registrations between the native anatomical space and the INIA19 template image^46^, so that the macaque anatomy could be appropriately indicated using the associated NeuroMaps labelling.

First, the intensity bias field of each image was corrected using the “N4BiasFieldCorrection” tool in ANTS^47^.Next, the T_1_-weighted images for the first imaging session for each macaque were B-spline nonlinearly registered to the full head image from the INIA19 template using “antsRegistrationSyN.sh”^48^. The output from that transformation was then used to map the INIA19 brain mask to each macaque’s native space using a nearest neighbor interpolation method.

For any existing subsequent images for each macaque, the brain masks were generated using a merged transformation incorporating the nonlinear registration to the INIA19 template from the first session and a chain of linear registrations to the native space images of the same macaque at the previous time point. These resulting brain masks were used to skull-strip all the anatomical images. Next, the process of intensity bias correction for all the skull-stripped brain images was repeated using the obtained brain masks to limit the correction region and improve the quality of the corrections.

After these preprocessing steps, the brain images were coregistered to the INIA19 brain template for the purpose of applying the NeuroMaps labels to anatomy in the macaque’s native space. To begin, all first session brain images were nonlinearly registered to the INIA19 brain template. Using the transformation output, the NeuroMaps label map was inversely mapped to each macaque’s native space using nearest neighbor interpolation. Once again, the coregistration procedure for subsequent sessions proceeded by concatenating linear transformations with the brains from previous imaging sessions and the non-linear transformation from the first session with the INIA19 brain template. Using this procedure, label maps were generated for each image.

Two images were discarded due to the inability to coregister the images to the INIA19 template using a consistent protocol. The final imaging dataset included 14 first session scans, 8 s session scans, 1 third session scan, and 1 fourth session scan.

The volumetric analysis was based on the sizes of the regions within the label maps for each macaque. Because apoE levels were analyzed in the prefrontal cortex (PFC), amygdala (AMYG), and hippocampus (HIPP), we defined three well-resolved regions-of-interest (ROIs) from the smaller, bilateral parcellations included in the NeuroMaps labels comprising these regions. The ROI boundaries were defined a priori before any statistical analysis of the volumes and are equivalent to those used in previous work^15^. To correct for differences in head size, the ROIs were normalized using the total brain volume, and these relative volumes were used for all subsequent analyses.

### Statistical analysis

All statistical analyses were completed using the computing environment *R* (version 4.0.3)^49^. *R* packages used for the analysis and presentation of the data included: psych (2.0.9), xlsx (0.6.5), tidyverse (1.3.0), geepack (1.3.1), corrplot (0.84), and ggplot2 (3.3.2)^50–57^.

Our a priori hypothesis was that regional levels of apoE may be related to regional relative volumes. ApoE levels may be related to regional relative volumes in the same or non-overlapping regions. To explore this, we examined the correlation matrix of regional apoE levels and regional relative volumes from the images in the first session. We set a liberal threshold *p* < 0.20, in order to identify correlations to explore further using generalized estimating equations (GEE)^58^. GEE was chosen due to the unique features of the Wake Forest Radiation Survivor Cohort sample, which involves data of macaques used across different studies at six different institutions and imaged at different times at Wake Forest. The use of GEE allowed us to incorporate longitudinal data where it was acquired, even though there were not sufficient longitudinal data to justify exploring changes over time. Thus, GEE could give us estimated effects of apoE levels and radiation exposure in predicting regional volumes, while incorporating all of the images acquired as part of the sample. We tested the effects of regional apoE levels as distinct models because the sample size was small and apoE levels could not be quantified for all the regions in each animal. As a secondary hypothesis, we also tested whether radiation dosage predicted the age of the macaques at death.

In order to control the Type I error rate, we set the false discovery rate (FDR) at $\alpha = 0.0{5}$ by using the Benjamini-Hochberg (BH) procedure^59^, using the number of correlations tested in our exploratory procedure, the tests of radiation dosage on age, and apoE levels in the three brain regions (*m* = 19) as the number of comparisons. The BH corrected *p*-values for the effects of radiation dosage and regional apoE levels are provided in the results.

## Results

**Figure 2:**
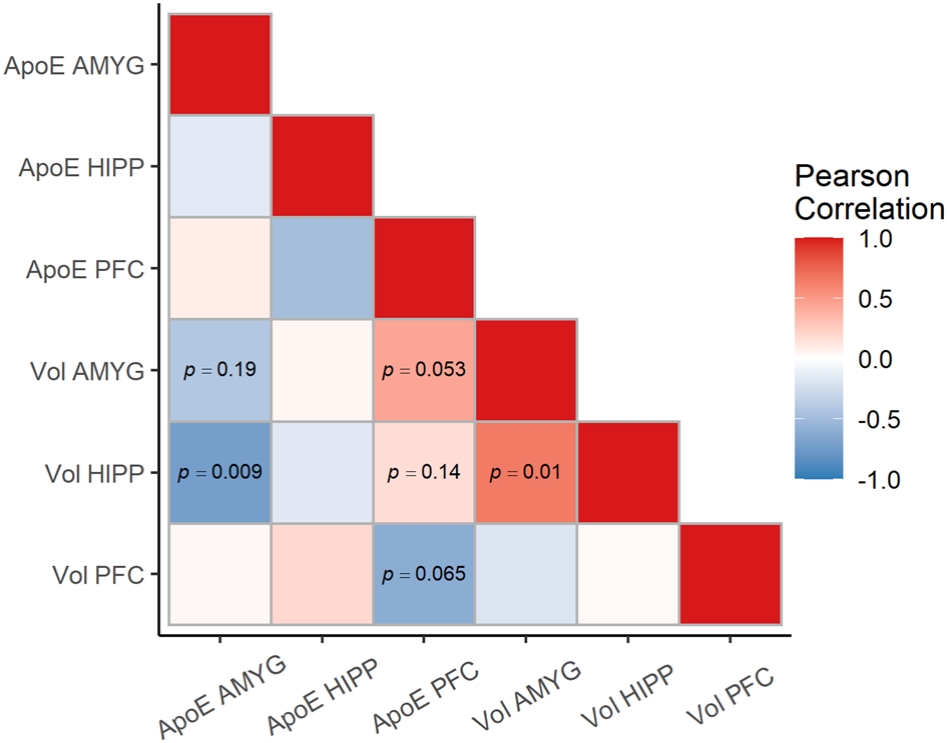
Correlation matrix of the regional apoE levels in the amygdala (AMYG), hippocampus (HIPP), and prefrontal cortex (PFC) and the relative volumes of those regions. Correlations meeting a *p* < 0.20 threshold are shown in the correlation matrix and were used for subsequent analysis.

Using the first session of imaging data for each macaque, we conducted an exploratory analysis of the correlations between regional apoE levels and regional relative brain volumes (Fig. 2). Based on this analysis, we identified the regional apoE levels to use as predictors, along with radiation, to predict the regional relative volumes in the brain. These included using apoE levels in the PFC to predict relative PFC volumes, both AMYG and PFC apoE to levels predict relative AMYG volumes, and both AMYG and PFC apoE levels to predict relative HIPP volumes. We also assessed if HIPP apoE levels predicted HIPP volumes, since we had an a priori hypothesis of regional relatedness. Interestingly, we also found that the relative volumes of the hippocampus and amygdala were moderately correlated *r*(12) = 0.66, *p* = 0.01 (uncorrected).

Using GEE, we modeled the relative regional volumes as dependent variables and used regional ApoE levels and radiation dosage as independent variables. The initial working correlation structure was assumed to be autoregressive (lag 1; AR1), due to the longitudinal aspect of data acquired from the same animal.

**Figure 3:**
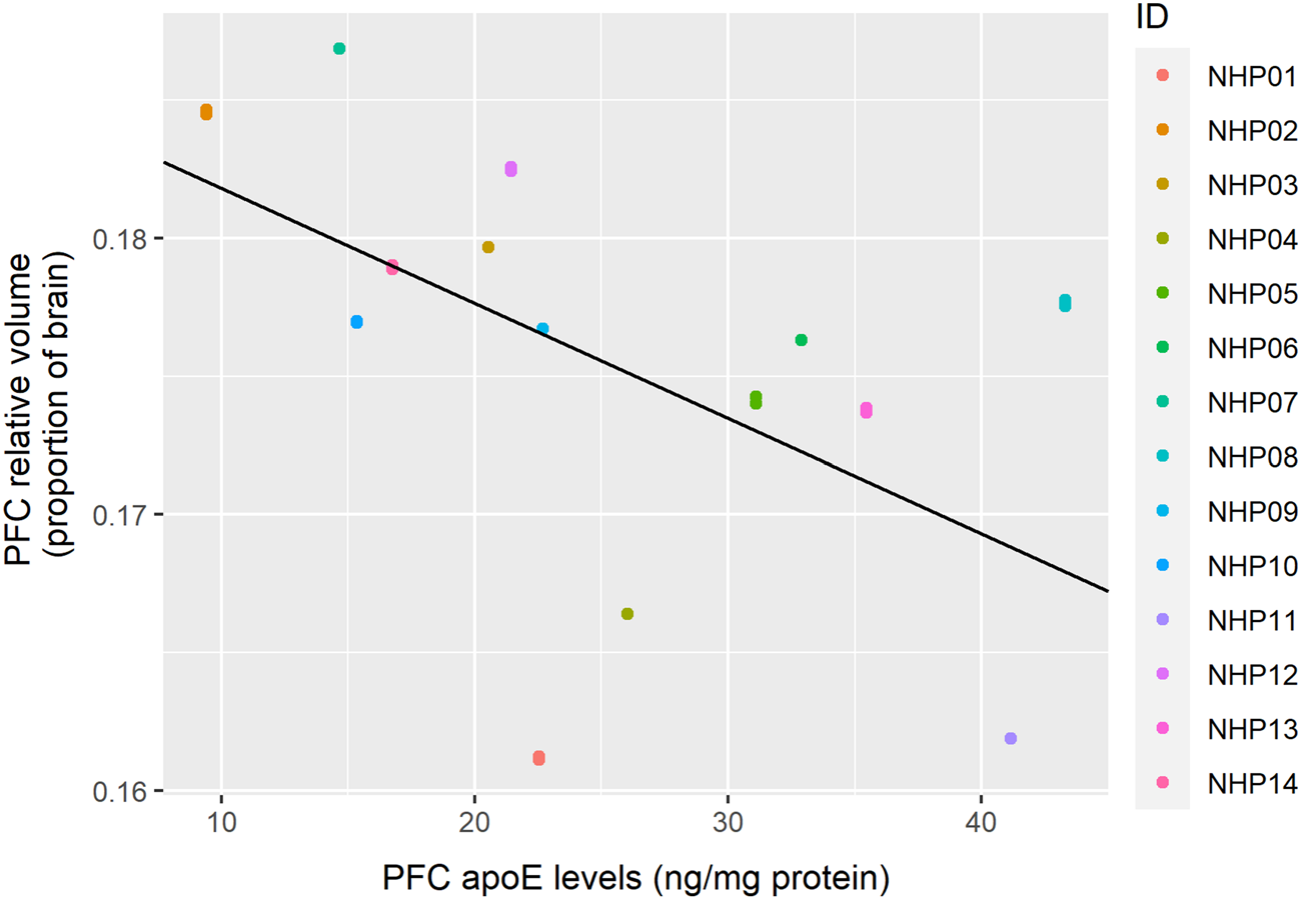
Levels of apoE in the PFC negatively predicts relative PFC volume. The figure shows results from a GEE model using PFC apoE as the sole predictor. Individual macaque IDs are distinctly colored.

The relative volume of the PFC was significantly predicted by PFC apoE levels (Table 3, Fig. 3; corrected *p* = 0.025). Greater PFC apoE levels predicted lower relative PFC volume. The effect of radiation dose did not significantly predict PFC volume after correcting for multiple comparisons (corrected *p* = 0.14).

**Table 3:** Linear generalized estimating equations model results predicting relative prefrontal cortex volumes with apoE in the PFC and radiation dose as independent predictors.

Fixed effects	Estimate	Standard error	Wald	Pr ( >|W|)
(Intercept)	2.30e−01	2.30e−02	9.96e+01	< 2.00e−16***
PFC ApoE levels	− 4.20e−04	1.50e−04	8.18e+00	4.20e−03**
Radiation Dose (gy)	− 6.60e−03	3.50e−03	3.56e+00	5.90e−02^+^

***p < 0.001; **p < 0.01; ^+^*p* < 0.1; displayed *p*-values are uncorrected for multiple comparisons.

**Figure 4:**
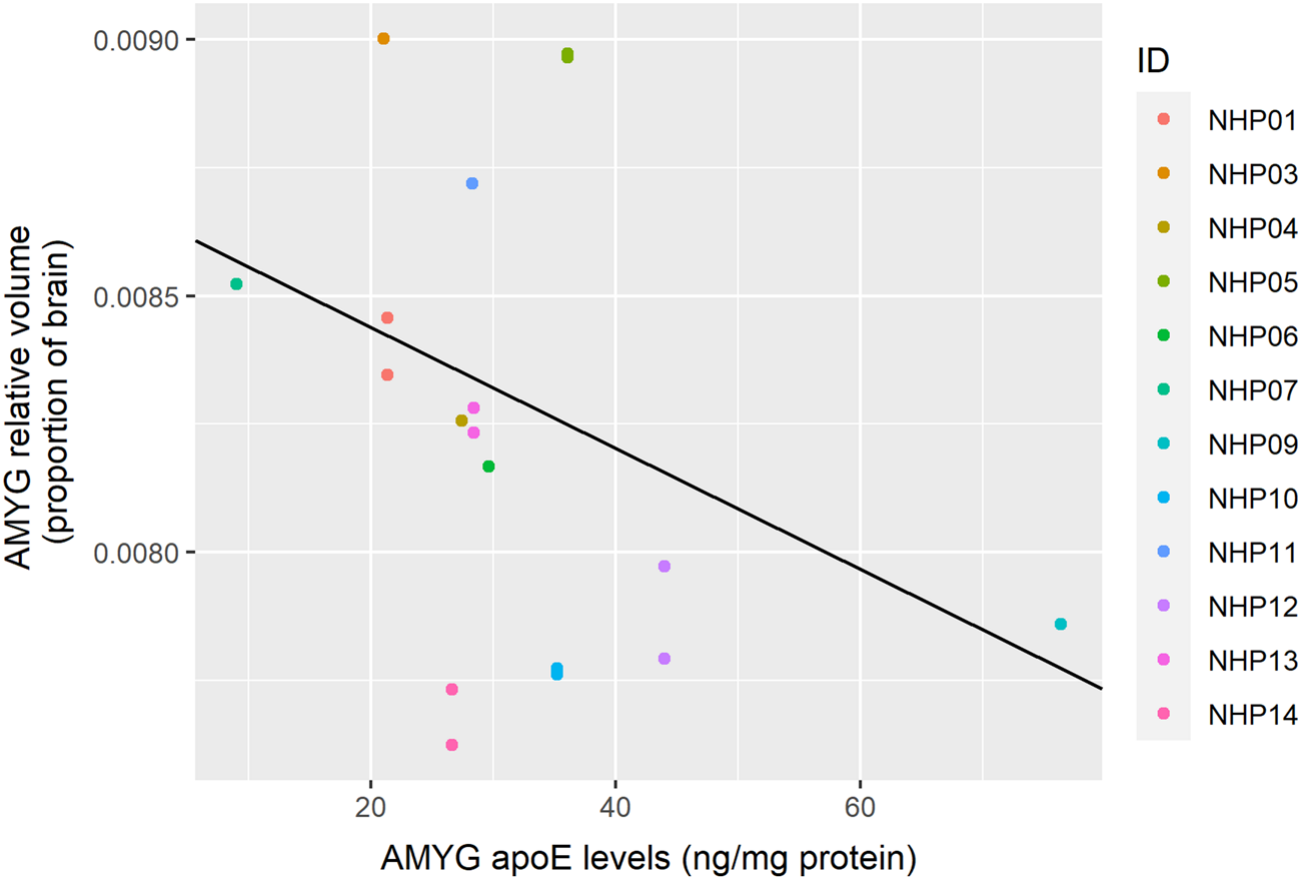
Levels of apoE in the AMYG negatively predicts relative AMYG volume. The figure shows results from a GEE model using AMYG apoE levels as the sole predictor. Individual macaque IDs are distinctly colored.

**Figure 5:**
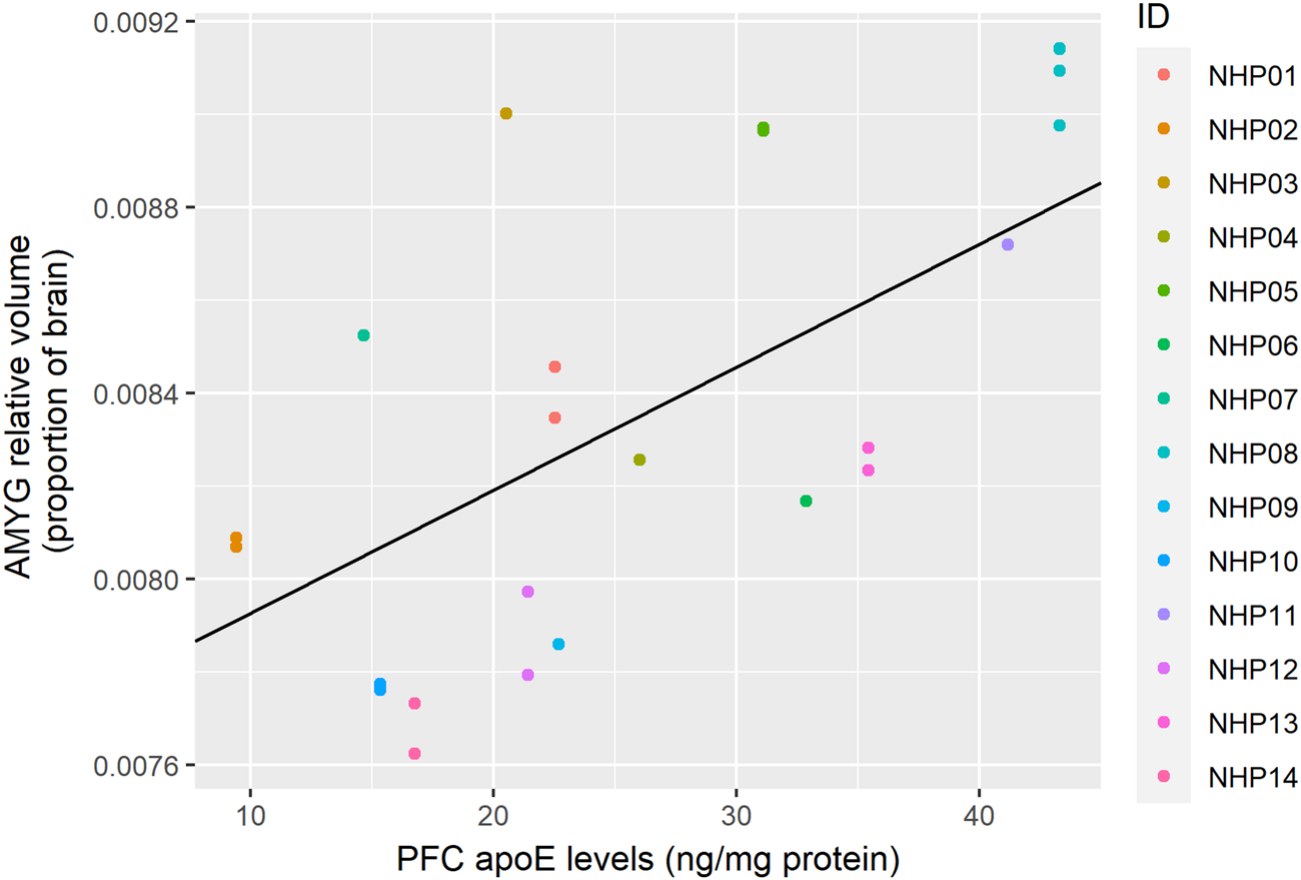
Levels of apoE in the PFC positively predicts relative AMYG volume. This is the opposite effect of the AMYG apoE on relative AMYG volume. The figure shows results from a GEE model using PFC apoE levels as the sole predictor. Individual macaque IDs are distinctly colored.

Relative AMYG volume was predicted by both AMYG apoE levels (Table 4, Fig. 4; corrected *p* = 0.0039) and PFC apoE levels (Table 5, Fig. 5; corrected *p* = 0.0079). However, there was anatomical specificity regarding the direction of change; greater AMYG apoE levels predicted lower AMYG relative volume, whereas greater PFC apoE levels predicted higher AMYG relative volumes. In neither model did radiation dose predict relative AMYG volume.

**Table 4:** Linear generalized estimating equations model results predicting relative amygdala volumes with ApoE levels in the amgydala and radiation dose as independent predictors.

Fixed effects	Estimate	Standard error	Wald	Pr (>|W|)
(Intercept)	1.03e−02	1.20e−03	7.30e+01	< 2.00e−16***
Amgydala ApoE levels	− 1.18e−05	3.17e−06	1.38e+01	2.07e−04***
Radiation dose (Gy)	− 2.31e−04	1.68e−04	1.89e+00	1.70e−01

***p < 0.001; displayed *p*-values are uncorrected for multiple comparisons.

**Table 5:** Linear generalized estimating equations model results predicting relative amygdala volumes with ApoE in the PFC and radiation dose as independent predictors.

Fixed effects	Estimate	Standard error	Wald	Pr (>|W|)
(Intercept)	9.40e−03	1.31e−03	5.12e+01	8.40e−13***
PFC ApoE levels	2.59e−05	7.76e−06	1.12e+01	8.31e−04***
Radiation Dose (Gy)	− 2.46e−04	1.84e−04	1.78e+00	1.82e−01

***p < 0.001; displayed *p*-values are uncorrected for multiple comparisons.

**Figure 6:**
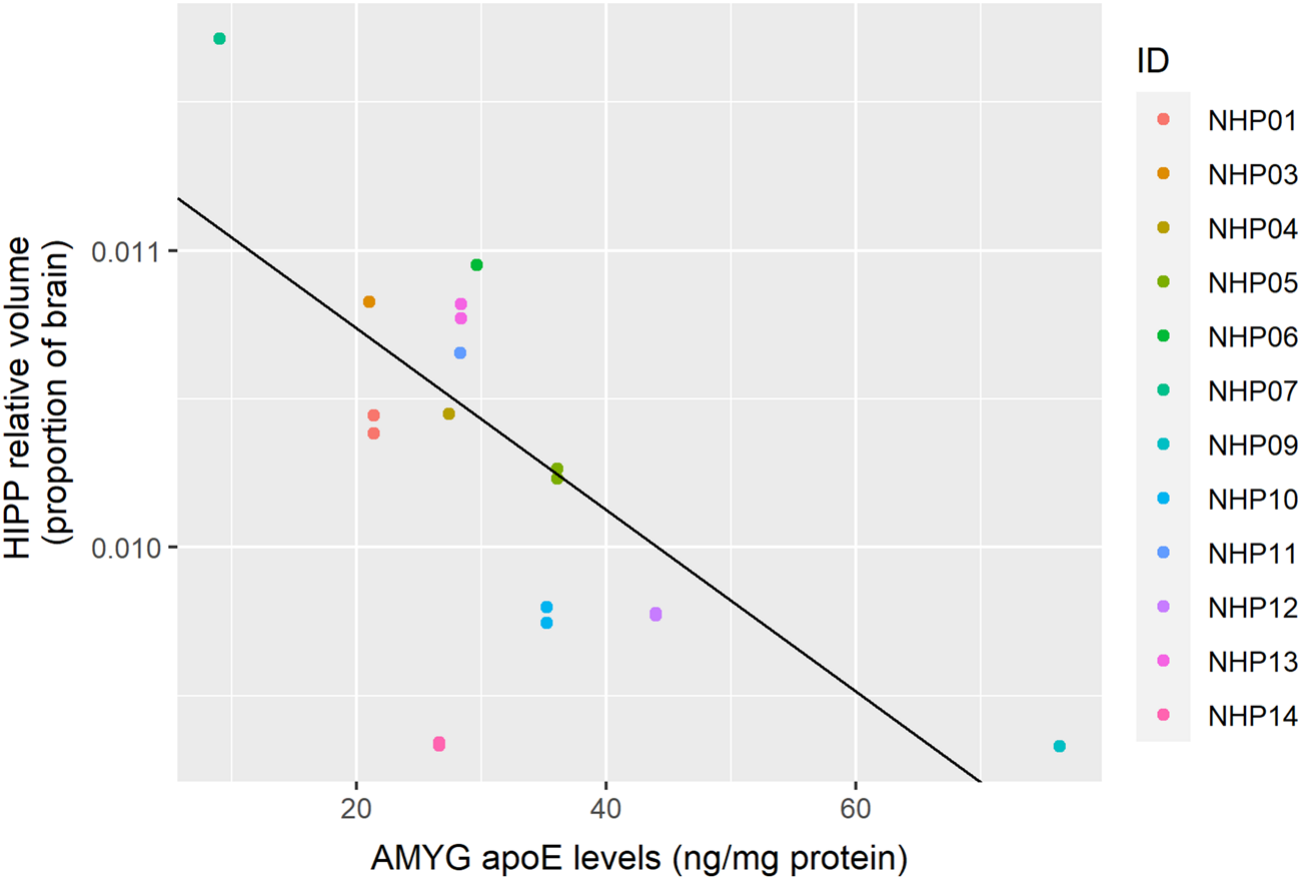
Levels of apoE in the AMYG negatively predicts relative HIPP volume. The figure shows results from a GEE model using HIPP apoE levels as the sole predictor. Individual macaque IDs are distinctly colored.

**Figure 7:**
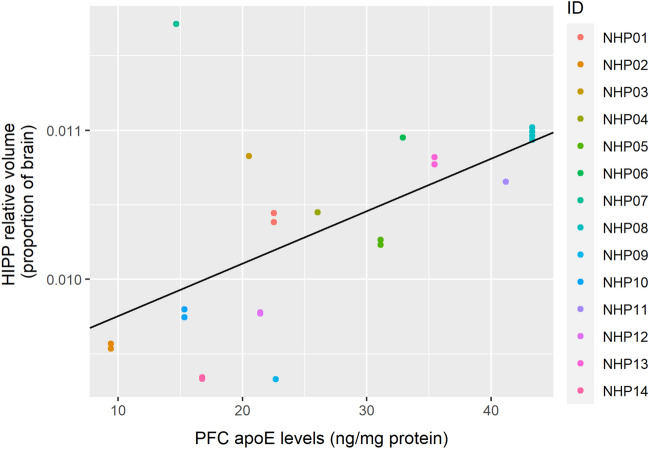
Levels of apoE in the PFC positively predicts relative HIPP volume. This is the opposite effect of the AMYG apoE levels on relative HIPP volume. The figure shows results from a GEE model using PFC apoE levels as the sole predictor. Individual macaque IDs are distinctly colored.

The relative HIPP volume was not significantly predicted by HIPP apoE levels (Table 6). However, it was predicted by AMYG apoE levels (Table 7; Fig. 6; corrected *p* = 0.041) and by PFC apoE levels (Table 8; Fig. 7; corrected *p* = 0.032). Similarly to the AMYG volumes, greater AMYG apoE levels predicted lower relative HIPP volumes, and greater PFC apoE levels predicted larger relative HIPP volumes. Interestingly, radiation dosage also significantly predicted relative HIPP volumes in the model using AMYG apoE levels as a co-predictor (corrected *p* = 0.025), but not when using HIPP apoE levels (corrected *p* = 0.32) or PFC apoE levels (corrected *p* = 0.64). Greater radiation dosage predicted lower relative HIPP volume. Because radiation dosage did not predict the relative volumes in the other models, we examined the correlations between radiation dosage and apoE in our three ROIs. All of these correlations were non-significant (AMYG apoE: *r*(10) = − 0.0074, corrected *p* = 1.0; HIPP apoE: *r*(11) = − 0.19, corrected *p* = 0.73; PFC apoE: *r*(12) = − 0.036, corrected *p* = 1.0).

**Table 6:** Linear generalized estimating equations model results predicting relative hippocampal volumes with ApoE levels in the hippocampus and radiation dose as independent predictors.

Fixed effects	Estimate	Standard Error	Wald	Pr ( >|W|)
(Intercept)	1.34e−02	2.20e−03	3.69e+01	1.22e−09***
Hippocampal ApoE levels	− 8.07e−06	1.27e−05	4.00e−01	5.27e−01
Radiation Dose (Gy)	− 3.98e−04	3.00e−04	1.75e+00	1.85e−01

***p < 0.001; displayed *p*-values are uncorrected for multiple comparisons.

**Table 7:** Linear generalized estimating equations model results predicting relative hippocampal volumes with ApoE levels in the amgydala and radiation dose as independent predictors.

Fixed effects	Estimate	Standard Error	Wald	Pr ( >|W|)
(Intercept)	1.39e−02	9.57e−04	2.11e+02	< 2.00e−16***
Amgydala ApoE levels	− 3.06e−05	1.23e−05	6.18e+00	1.29e−02*
Radiation dose (Gy)	− 3.61e−04	1.29e−04	7.82e+00	5.17e−03**

***p < 0.001, **p < 0.01, *p < 0.05; displayed *p*-values are uncorrected for multiple comparisons.

**Table 8:** Linear generalized estimating equations model results predicting relative hippocampal volumes with ApoE levels in the PFC and radiation dose as independent predictors.

Fixed effects	Estimate	Standard Error	Wald	Pr ( >|W|)
(Intercept)	1.10e−02	2.17e−03	2.58e+01	3.76e−07***
PFC ApoE levels	3.30e−05	1.25e−05	6.96e+00	8.34e−03**
Radiation dose (Gy)	− 2.19e−04	2.65e−04	6.86e−01	4.08e−01

***p < 0.001, **p < 0.01; displayed *p*-values are uncorrected for multiple comparisons.

Although our main interest was in predicting the relative regional volumes after correcting for total brain volume, results for models predicting the total volumes are included as supplemental tables (Supplemental Tables 1–3). Here we only used the corresponding regional apoE levels as an independent variable to predict regional brain volumes.

**Figure 8:**
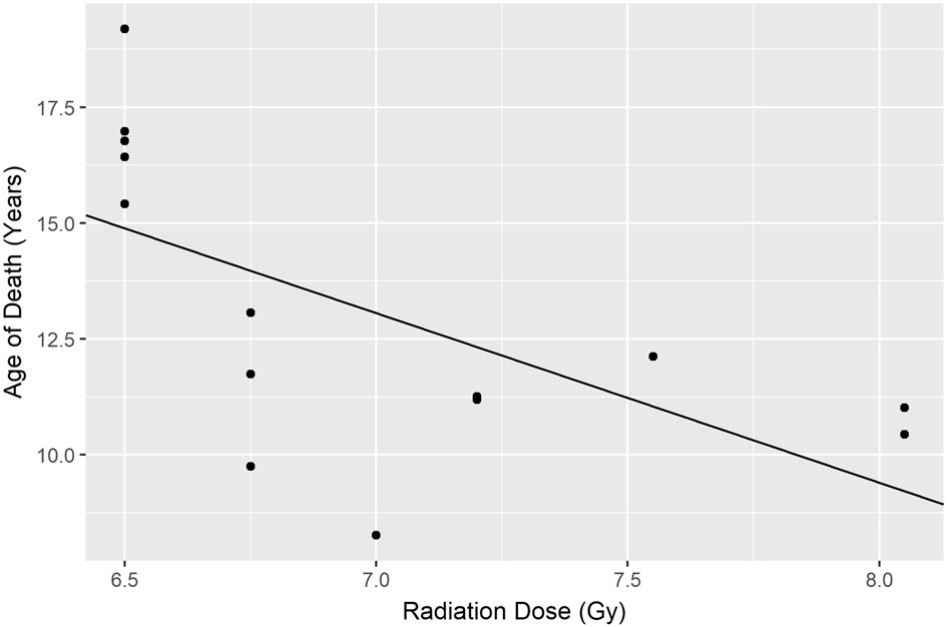
Larger dosages of radiation predict a younger age at necropsy.

Finally, we tested the hypothesis that radiation dosage predicts the age at death of the macaque, consistent with the idea that radiation exposure may model accelerated aging. Radiation dose negatively predicted the age of the macaque at death *r*(12) =  − 0.63, corrected *p* = 0.046), implying that macaques receiving higher doses of radiation lived less long (Fig. 8).

As a supplementary analysis, we investigated whether levels of α-klotho in the amygdala or PFC were associated with any of our outcome measures. We found that protein levels of α-klotho in the amygdala and PFC did not predict any regional brain volumes, or whole brain volume. Levels of α-klotho were also not correlated with radiation dose. Levels of α-klotho also did not change with age in this sample. We did however find a strong relationship between α-klotho and apoE protein levels in the amygdala *r*(10) = 0.80, *p* = 0.002 (Supplementary Figure 1). This relationship did not exist in the PFC.

## Discussion

In the present study, we observed a regionally specific separation between limbic and frontal regions in terms of how well regional apoE levels predict relative brain volumes. Levels of apoE in the amygdala correlated negatively with relative volume of both the amygdala and the hippocampus, but not relative volume of the PFC. Similarly, apoE levels in the PFC negatively correlated with the relative PFC volume. However, in contrast to this, apoE levels in the PFC correlated positively with relative size of the amygdala and hippocampus. Interestingly, amygdala apoE, but not hippocampal apoE, levels predicted relative hippocampal volume. It is possible that increased apoE levels in the amygdala drove changes in both of these limbic regions due to their interconnectedness. For example, amyloid β (Aβ), generated from amyloid precursor protein, has been shown to spread trans-synaptically, with increased spread in highly interconnected regions^60^. ApoE is involved in the metabolism, aggregation, deposition, and clearance of Aβ and binds to it, thus this could be a factor in the results we observed^28^.

The question remains, why does increased apoE levels within regions predict smaller relative volumes of those and other regions? The effects of apoE in the brain are still being elucidated. Low apoE levels are associated with poorer cognitive outcomes, and homozygous apoE4 carriers have lower apoE levels in the brain than homozygous apoE3 or apoE2 carriers, as well as increased vulnerability to AD^22,23,28^. However, it is unclear if this relationship is secondary to lower apoE levels reflecting more apoE bound in Aβ plaques, the cellular origin of the apoE, or if apoE is playing a protective compensatory role in the brain and is upregulated by environmental stressors. ApoE levels in the PFC and hippocampus also increase with age in both rodents and macaques^27^. Our mouse studies indicate that apoE levels are modulated in a non-linear dose–response curve four months following Helium ion (250 MeV/n; 0.21, 0.42 or 1.68 Gy) IR in B6D2F1 mice and increased within two months following^56^Fe ion (600 MeV/n, 0.5 Gy) irradiation in brains of human apoE3, but not of apoE2 or apoE4, transgenic mice, suggesting an adaptive role for apoE^25,26^. Thus, we investigated whether regional apoE levels in this study were correlated with the dose of radiation. We did not find an association between the two, suggesting that apoE levels either are not altered in response to IR in macaques, in contrast to our rodent results, or that the changes in brain apoE levels are more transient and not seen following longer intervals between radiation and assessments of the brain levels. We also recognize that age-related increase in brain levels of apoE might have masked radiation-induced modulation of apoE levels.

Additionally, we found that apoE levels in the PFC positively correlated with relative hippocampus and amygdala volume. Relative PFC volume was not anticorrelated with relative hippocampus or amygdala volume, thus the relationship seems specific to apoE levels. While the PFC and limbic system are known to be extensively bidirectionally connected, it is not clear why apoE levels in the PFC predicted larger relative hippocampus and amygdala volumes in this study. One possibility is the inhibitory function of the PFC. The PFC is known to exhibit inhibitory control over the amygdala, such that increased PFC activity during a task of emotional regulation is correlated with decreased amygdala activity^61^. The PFC also exercises top-down modulation of hippocampal activity in retrieval suppression^62^. Thus, it is possible that an increase in apoE in the PFC functions to disinhibit these limbic areas, resulting in a volumetric increase.

In a supplementary analysis, we investigated whether levels of α-klotho in the PFC and amygdala were related to levels of apoE in those areas. We found a robust relationship between levels of apoE and α-klotho in the amygdala, but no relationship in the PFC. Higher levels of α-klotho are neuroprotective, and this strong region-specific positive relationship between apoE and α-klotho suggests that the observed increase in apoE was an adaptive response to the environmental stress of radiation exposure.

We found that within the limitation of the radiation doses involved in this study that radiation dose bore a strong negative correlation with age, suggesting that macaques receiving higher doses of radiation did not live as long. We recognize that this relationship may vary at doses outside of the range examined in this study. There may be a threshold dose below which radiation does not bear a correlation with lifespan. The exposures in this study were systemic, and thus morbidity and mortality may be due to extra-CNS effects.

We found no effect of radiation on total brain volume. A review of dose-volume effects of clinical radiation exposure on the brain found that a cumulative 120 Gy dose of IR poses a 5% risk of radiation-induced necrosis with a median onset of necrosis 1–2 years after radiation therapy50. The doses of radiation included in this study were well below that, at 6.5–8.05 Gy. Thus, it is in accordance with previous literature that we did not observe necrosis or gross reduction in brain volume.

We did not observe a direct relationship between radiation dose and relative volume of the PFC or amygdala. It is possible that radiation dose does affect regional volumes of these areas, but that these changes were too subtle to be detected in our study given our limited sample size and large inter-subject variability. We did find that higher doses of radiation were associated with decreased relative hippocampal volume when controlling for amygdala apoE levels. This was not found when using hippocampal apoE levels or PFC apoE levels as co-predictors. This suggests that the portion of the variance shared between radiation dose and amygdala apoE levels positively predicts the relative size of the hippocampus. In other words, in the context of radiation, apoE may have a protective compensatory effect on regional brain volume. Future experimental research should specifically target this hypothesis.

There were some limitations inherent in the work presented here. We utilized archived, previously collected data from a cohort of irradiated Rhesus macaques, limited to subjects that came to necropsy, within the unique RSC at Wake Forest University in North Carolina. Thus, we did not analyze an age-matched, non-irradiated control group. This group would have allowed us to compare the effects of apoE levels on brain volume with and without irradiation. The macaques received variable doses of radiation and variable environmental conditions in different experiments at six distinct institutions investigating the effects of IR before they were transferred for long-term observation within the RSC. Thus, we were limited by a relatively small sample size as well as different historical environmental conditions, likely contributing to a relatively high inter-subject variability.

In this study, we were underpowered to investigate how irradiation affects brain volume over time as only a subset of macaques underwent multiple scanning sessions. Previous studies have shown that factors such as increasing age, impaired glycemic control, and uncontrolled hypertension can all worsen radiation effects on the brain^63^. Thus, these factors could have contributed enough variance to mask existing dose-dependent effects of radiation on regional brain volumes and apoE levels. Future efforts are warranted using a larger sample size and multiple time points to investigate changes over the course of several years.

This study included only males, and previous research shows that radiation effects on the brain can be sex-dependent^24^. Female macaques were not available for inclusion at the time of necropsy in the present study, but future work should include both sexes to investigate any sex-dependent effects of radiation on the CNS.

Radiation dose–response curves with regard to brain-related outcome measures are often complex and not linear, perhaps due to threshold effects and lack of compensatory mechanisms at lower doses^25^. Thus, future studies are warranted to expand the number of animals given a greater range of doses of radiation to more fully understand the relationship between radiation dose and brain health.

In summary, regional apoE levels predicted regional volume in the amygdala and the prefrontal cortex. In addition, apoE levels in the amygdala, but not the hippocampus, strongly predicted relative hippocampal volume. Finally, radiation dose negatively affected relative hippocampal volume when apoE in the amygdala is controlled for, indicating a possible protective role of regional apoE in the context of radiation exposure. The associations between regional apoE levels and relative brain volumes, as well as the finding that the relative size of the PFC and amygdala might be spared from radiation-induced atrophy, is of high translational relevance to human health. ApoE might protect selected brain areas from radiation-induced injury and atrophy. This work contributes to our understanding of the effects of IR in the primate brain, as well as the role of apoE in the irradiated brain, and could inform future therapies to mitigate the adverse effects of IR on the CNS.

## Supplementary Information

Supplementary Information.

## Data Availability

The datasets generated during and/or analyzed during the current study are available from the corresponding author on reasonable request.
