# Supplementary material for: Apolipoprotein E levels in the amygdala and prefrontal cortex predict relative regional brain volumes in irradiated Rhesus macaques
Source: Sci Rep. 2021 Nov 11;11:22130. doi: 10.1038/s41598-021-01480-3 (PMC8585884; doi:10.1038/s41598-021-01480-3)
Supplement: Supplementary file 1 — Supplementary Information. [file 41598_2021_1480_MOESM1_ESM.docx]

Apolipoprotein E levels in the amygdala and prefrontal cortex predict relative regional brain volumes in irradiated Rhesus macaques

# Payel Kundu1+, Benjamin Zimmerman1,2,3+, Ruby Perez1, Christopher T. Whitlow4, J. Mark Cline4,5, John D. Olson5, Rachel N. Andrews4,5, Jacob Raber1,6*

^1^Department of Behavioral Neuroscience, Oregon Health and Science University, Portland, OR, USA

^2^Advanced Imaging Research Center, Oregon Health and Science University, Portland, OR, USA

^3^Beckman Institute for Advanced Science and Technology, University of Illinois at Urbana- Champaign, Urbana, IL, USA

^4^ Department of Radiation Oncology, Wake Forest University, Wake Forest University School of Medicine, Winston-Salem, NC, USA

^5^ Department of Pathology, Section on Comparative Medicine, Wake Forest University School of Medicine, Winston-Salem, NC, USA

^6^Departments of Neurology and Radiation Medicine, Division of Neuroscience, ONPRC, Oregon Health and Science University, Portland, OR, USA

# + These authors contributed equally to the manuscript.

*** Correspondence:** Corresponding Author [raberj@ohsu.edu](mailto:raberj@ohsu.edu)

**Supplementary Table 1.** Linear generalized estimating equations model results predicting raw prefrontal cortex volumes with apoE levels in the PFC and radiation dose as independent predictors.

| **Fixed effects** | **Estimate** | **Standard Error** | **Wald** | **Pr (>IWI)** |
| --- | --- | --- | --- | --- |
| (Intercept) | 2.36e+4 | 6.18e+03 | 1.46+01 | 1.30e-04*** |
| PFC apoE levels | -1.28e+02 | 4.16+01 | 9.50e+00 | 2.05e-03** |
| Radiation Dose (Gy) | -1.lle+02 | 7.95+02 | 2.00e-02 | 8.89e-01 |

****p* < 0.001, ***p* < 0.01; displayed p-values are uncorrected for multiple comparisons

**Supplementary Table 2.** Linear generalized estimating equations model results predicting raw amygdala volumes with apoE levels in the amgydala and radiation dose as independent predictors.

| **Fixed effects** | **Estimate** | **Standard Error** | **Wald** | **Pr (>IWI)** |
| --- | --- | --- | --- | --- |
| (Intercept) | 8.50e+02 | 2.42e+02 | 1.24e+0l | 4.40e-04*** |
| Amygdala apoE levels | -8.20e-01 | 3.8le+00 | 5.00e-02 | 8.30e-01 |
| Radiation Dose (Gy) | 1.4le+0l | 3.05e+0l | 2.l0e-01 | 6.45e-01 |

**** p* < 0.001; displayed p-values are uncorrected for multiple comparisons

**Supplementary Table 3.** Linear generalized estimating equations model results predicting raw hippocampal cortex volumes with apoE levels in the hippocampus and radiation dose as independent predictors.

| **Fixed effects** | **Estimate** | **Standard Error** | **Wald** | **Pr (>IWI)** |
| --- | --- | --- | --- | --- |
| (Intercept) | 1.14e+03 | 3.77e+02 | 9.13e+00 | 2.50e-03** |
| Hippocampal apoE levels | -1.54e+00 | 2.60e+00 | 3.50e-01 | 5.54e-01 |
| Radiation Dose (Gy) | 7.30e+00 | 4.74e+0l | 2.00e-02 | 8.78e-01 |

***p* < 0.01; displayed p-values are uncorrected for multiple comparisons


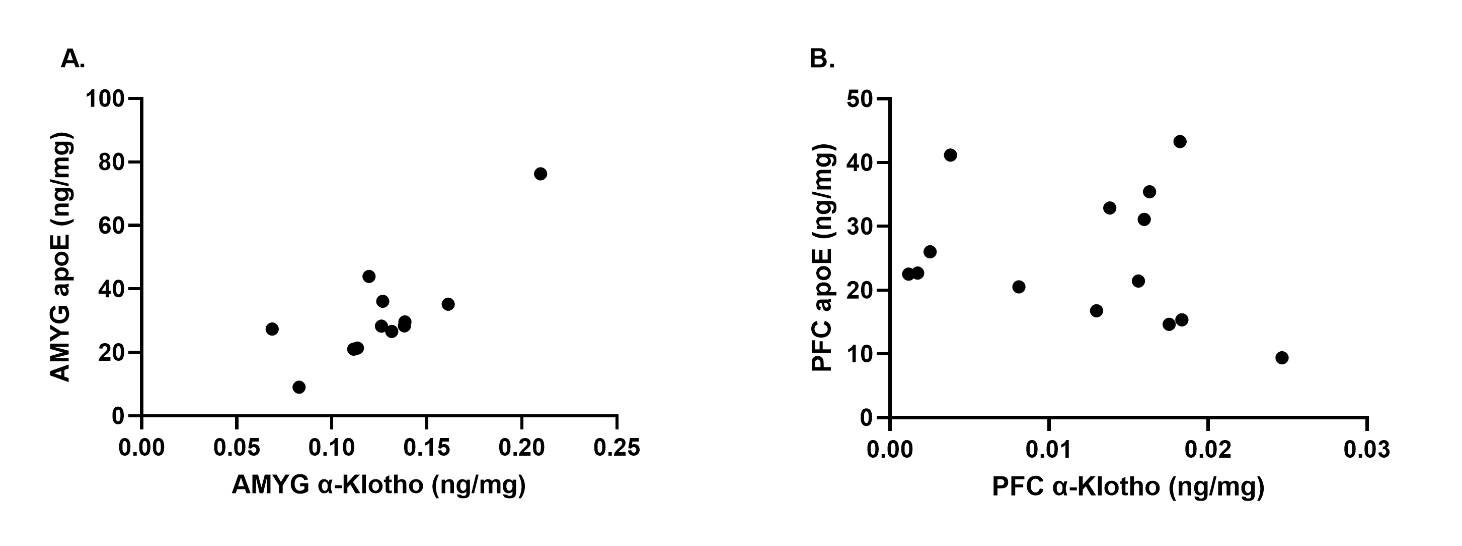


**Supplementary Figure 1.** Relationship of apoE and a-Klotho levels in the amygdala (AMYG) (**A**) and prefrontal cortex (PFC) (**B**). In the amygdala, levels of apoE and α-Klotho are highly correlated. This relationship does not exist in the PFC.
